# Sensitivity to linguistic register in 20-month-olds: Understanding the register-listener relationship and its abstract rules

**DOI:** 10.1371/journal.pone.0195214

**Published:** 2018-04-09

**Authors:** Ayaka Ikeda, Tessei Kobayashi, Shoji Itakura

**Affiliations:** 1 Graduate School of Letters, Kyoto University, Kyoto, Japan; 2 Japan Society for the Promotion of Science Research Fellow, Chiyoda-ku, Tokyo, Japan; 3 NTT Communication Science Laboratories, Nippon Telegraph & Telephone Corporation, Kyoto, Japan; Central European University, HUNGARY

## Abstract

Linguistic register reflects changes in speech that depend on the situation, especially the status of listeners and listener-speaker relationships. Following the sociolinguistic rules of register is essential in establishing and maintaining social interactions. Recent research suggests that children over 3 years of age can understand appropriate register-listener relationships as well as the fact that people change register depending on their listeners. However, given previous findings that infants under 2 years of age have already formed both social and speech categories, it may be possible that even younger children can also understand appropriate register-listener relationships. The present study used Infant-Directed Speech (IDS) and formal Adult-Directed Speech (ADS) to examine whether 20-month-old toddlers can understand register-listener relationships. In Experiment 1, we used a violation-of-expectation method to examine whether 20-month-olds understand the individual associations between linguistic registers and listeners. Results showed that the toddlers looked significantly longer at a scene in which the adult was talked to in IDS than when the infant was talked to in IDS. In contrast, there was no difference when the adult and the infant were talked to in formal ADS. In Experiments 2 and 3, we used a habituation switch paradigm to examine whether 20-month-olds understand the abstract rule that a change of register depends on listeners rather than on speakers. Results showed that the toddlers looked significantly longer at the scene where the register rule was violated. The present findings provide new evidence that even 20-month-olds already understand that people change their way of speaking based on listeners, although their understanding of individual register-listener relationships is immature.

## Introduction

When we communicate with other people, we adjust aspects of our own behavior and appearance, such as our body position, gestures, speech, and clothes, as appropriate to the time, place, and opportunity. For example, when we go to a wedding party, we dress up in formal clothes and behave politely. When we go to a casual party, we wear casual clothes and speak casually. At the same time, we use unfamiliar people’s personal behavior to guess information about them such as their social rank, status, hometown, and their relationships. For example, we judge a person as having a high rank when he or she adopts an expansive posture, speaks loudly, and gazes directly at their social partners [1–3]. Because understanding and following these conventional rules helps us to establish and maintain our social relationships, acquisition of these rules is an important topic in developmental psychology.

One important means of adjusting situations in conformance with conventional rules is the way of speaking. Even in a single language, there are many ways of speaking, and we choose the appropriate one when we talk with others. When talking to older people, we may speak in a polite way by using honorific words or changing our choice of pronouns [4]. When we talk to infants, we speak slowly using high pitch and a large pitch range [5–6]. In sociolinguistic terms, this adjustment of speech based on listeners and situations is called the *register* of the language. A register is distinct from dialect, which is based on the speaker’s traits, such as geographic origin, social class, and gender [7]. Thus we can say that dialect is strongly related to the speaker, while a register is strongly related to the listener and the social situation which the speaker is in.

There is evidence that children use register, although sample sizes have been small. Previous research has shown that even 2-year-old toddlers change their way of speaking depending on the situations they are in, by using whispering, clarification, and exaggerated intonation [8]. Although it has been reported that children around 3 years of age can manipulate register by using phonological features of infant-directed speech (IDS; e.g., clarification) to their 1- or 2-year-old siblings, this has been observed in limited situations, such as prohibiting and directing in play [9,10]. Experimental studies using role-playing have shown that children’s use of register develops as they grow. By around 6 years of age, they can change lexical and morphosyntactic features (e.g., discourse markers, polite expressions) and use several registers, including IDS and adult-directed speech (ADS), appropriately and consistently depending on their listeners and the social situation [11–13]. Therefore, acquisition of the appropriate use of a register appears to require a long time in terms of development.

On the other hand, the comprehension of linguistic registers depending on the listener slightly precedes the use of it. Wagner, Greene-Havas, and Gillespie (2010) showed that English-speaking 5-year-olds were able to guess the listener’s social status based on the register, but 3- to 4-year-olds had more difficulty [14]. This has been confirmed in Japanese-speaking 4- to 6-year-old children [15]. However, when the speaker-listener relationship was clarified by adding social cues (e.g., the speaker’s age group), even English-speaking 3-year-olds were able to identify listeners based on the register. In addition, Spanish-speaking children, who are given more linguistic cues (e.g., pronoun choice) than English-speaking children, were better than English-speaking children at identifying listeners based on the register, and even 3-year-olds were able to do this [16]. These studies suggest that the presence of social and linguistic cues may lead younger children to successfully identify register.

Wagner et al. (2010) [14] suggested that there are three requirements for children’s mastery of register. The first is recognition of the acoustic, lexical, and syntactic characteristics of language that signal register. Previous studies have shown that 2-day-old infants can distinguish their native language from a foreign language [17–20], and infants below one month of age can also distinguish IDS from ADS [21–24]. The second prerequisite is knowledge of the status of the listener and the relationships between speakers and listeners, or speakers and the occasion, that determine the social situation. Infants have shown sensitivity to social categories that may provide cues to such relationships. For example, 14-month-olds change their imitations depending on the model’s age [25,26], 6- to 12-month-olds distinguish race [27,28], and 3- to 4-month-olds distinguish gender [29]. The third requirement for the mastery of register is proper associations between language characteristics and social situations. Recent studies have shown that younger children have some understanding of the relationships between speakers and languages: 6-month-olds associate other-race faces with a non-native language [30]. Furthermore, 20-month-olds find it unsurprising that people cannot communicate when their native languages are different [31]. These studies suggest that infants under 2 years of age already understand that people in different linguistic areas speak different languages and that the relationship between language, speaker and listener is important in communication. In other words, infants below the age of 2 years are likely to understand the relationship between language characteristics and social categories exemplified by a speaker.

In the present study, we asked if toddlers under 2 years of age understand that one person can speak in different ways based on the situation, even in the same language. More specifically, we examined whether 20-month-old toddlers can understand the appropriate relationship between register and the listener. Because the age range between 17 and 19 months is the approximate period at which a vocabulary spurt occurs, and speaking in two-word utterances begins at 2 years of age [32,33], the amount of linguistic communication increases dramatically at this age, and learning of the communication rules should become more important. Thus, examining the understanding of register before the age of 2 should reveal the developmental pathway of register acquisition and contribute to our knowledge of the development of communicative competence [34,35].

In Experiment 1, we examined whether Japanese-speaking 20-month-olds understand the individual associations of linguistic registers by testing their expectations that a person would belong to a specific age group based on the register. In Experiments 2 and 3, we tested toddlers’ more abstract understanding of register by examining the factors that they consider to be important determinants of speech change.

We chose to test IDS and formal ADS. IDS, the register used when talking to infants, includes several characteristic features, such as higher and greater variability in pitch, lengthening of vowels and pauses, specific words used only for infants (e.g., *doggy*), and shorter and less complex utterances compared to ADS [6]. An additional feature of Japanese IDS is case marker omission [36–38]. However, we need to keep in mind that recent studies have reported that some characteristics of IDS, such as pitch and speech rate, are highly variable between individuals [39]. ADS is the register used when talking to adults. We speak formally to people whom we are meeting for the first time or who have higher status or are more senior than us. For example, an English speaker may say “Can you~?” to friends but “Could you~?” to acquaintances, and Japanese speakers use words or phrases categorized as honorifics, humble and polite expressions that are markers of formality and respect to the listener [40]. Thus, the two registers of IDS and formal ADS differ in acoustic, lexical, and syntactic characteristics.

We chose these registers for two reasons. Firstly, preschoolers have been shown to be sensitive to these, and formal ADS is easier for them to understand than casual ADS [14,16]. Secondly, toddlers may be sensitive to IDS because they often hear it in their everyday lives, and they begin to use an IDS-like register from relatively early in childhood [9,10]. We studied 20-month-olds because they already understand the relationships between speakers and languages [31].

## Experiment 1

The purpose of Experiment 1 is to examine whether toddlers make particular assumptions about the relationship between register (IDS and formal ADS) and the listener. We used the violation-of-expectation method based on toddlers’ looking time to expected and unexpected events as an index to examine whether toddlers expect a listener to belong to a specific age group according to a speaker’s use of IDS or formal ADS.

When we follow sociolinguistic rules, IDS is considered to be speech directed at infants, while formal ADS is speech directed at a higher ranking person or people to whom we should show respect [41,42]. Thus, if 20-month-olds are aware of this register-listener relationship, their looking time will be longer in situations that violate the rule. That is, they will look longer when an adult is talked to using IDS than when an infant is talked to using IDS, and when an infant is talked to using formal ADS than when an adult is talked to using formal ADS.

### Materials and methods

#### Participants

All experiments were conducted in either Kyoto University or NTT Communication Science Labs from 11 August 2014 to 30 November 2015 and were approved by the ethics review boards of Unit for Advanced Studies of the Human Mind, Kyoto University (26-P-26). Participants’ native language was Japanese, and they lived in the Kansai region of Japan. We obtained written informed consent from the parents of toddlers after an explanation of the content and methods of the study were provided. Participants received a small monetary reward. Also, the individuals and their parents/guardians in this manuscript have given written informed consent (as outlined in the PLOS consent form) to publish these case details.

Experiment 1 was conducted at Kyoto University. Sixteen 20-month-olds (M = 1;8:0; range = 1;7:13–1;8:14) who were registered with the Kyoto University Infant Research Fellow Program participated in Experiment 1. An additional 3 toddlers participated in the study but were excluded from analysis because of fussiness and inattention.

#### Stimuli

**Fig 1:**
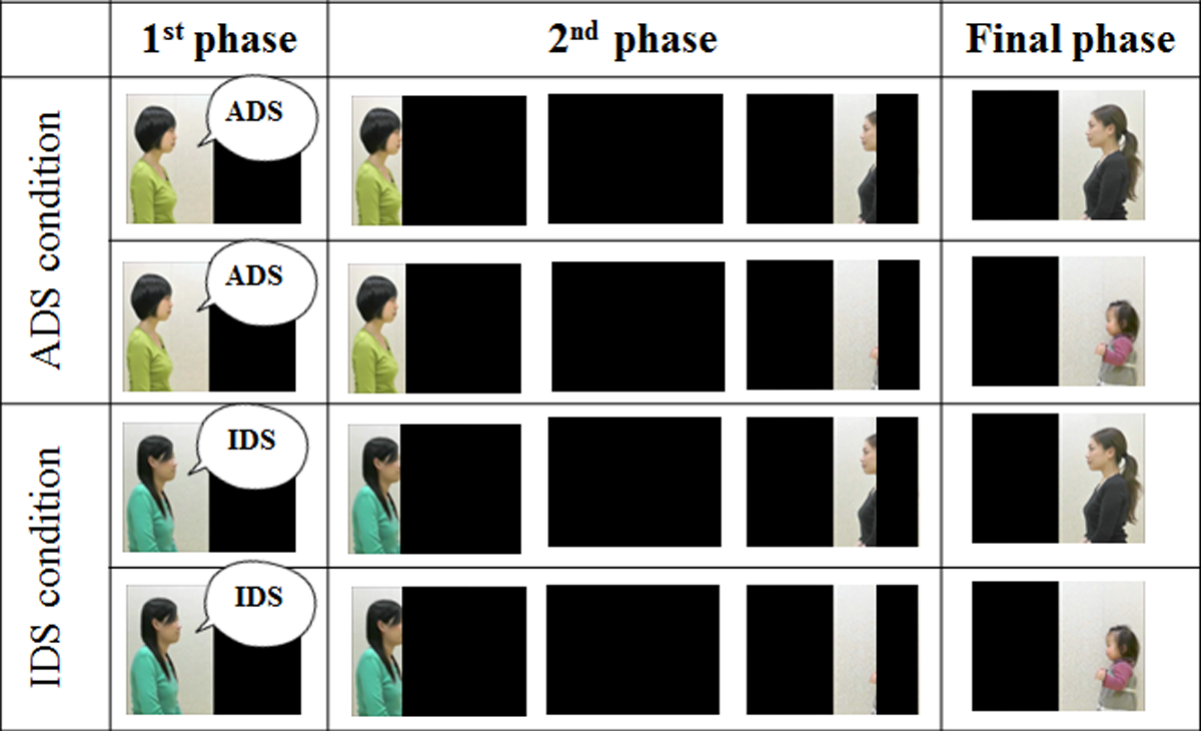
An example of stimulus presentation in test trials of Experiment 1. “IDS” indicates infant-directed speech and “ADS” indicates formal adult-directed speech. All toddlers participated in the pretest trials and two test conditions. After the pretest trials, ADS and IDS conditions were presented in semi-random order, such that the same register was not presented on more than 2 consecutive trials. In ADS/IDS conditions, after toddlers were shown that the speaker was talking to someone in IDS or ADS (10 sec), the black screen hid the speaker and the listener (infant or the adult) appeared.

Using Adobe After Effect (Adobe Creative Suite 5 Production Premium), we prepared 8 test movies, in which 2 persons, one speaker, and one listener, appeared at the same time. The speaker and listener were selected from 4 people: 2 Japanese women were speakers and a woman, and a 15-month-old girl were listeners. The movies consisted of 3 phases (Fig 1). In the first phase, the speaker faced the listener and spoke in IDS or formal ADS, but the listener was hidden from the participant behind a black screen. During this phase, the speaker gave a greeting and asked the listener’s name (10sec; IDS: “Konnichiwa, Kyo wa kokomade kitekurete arigato. Onamae oshietekurerukana?” [Hi, thanks for coming today. What’s your name?]; formal ADS: “Konnitiwa. Honjitsu wa kokomade okoshikudasari arigatougozaimasu. Onamae o oshiete itadakemasudesyouka?” [Hello, I appreciate your attendance today. Could you give me your name, please?]). The utterances in the two registers had different acoustic features: mean pitch was higher for IDS (M = 341.83 Hz) than for formal ADS (M = 291.63 Hz), pitch range was larger for IDS (M = 324.61 Hz) than for ADS (M = 275.59 Hz), and mean speech rate per mora was longer for IDS (M = 0.17 sec) than for formal ADS (M = 0.13 sec). In addition, IDS included case marker omission, a characteristic of Japanese IDS [36–38], and formal ADS involved humble and formal expressions, markers of formality and respect to the listener [40]. In the second phase, the black screen was moved to reveal the listener just after it moved to hide the speaker (2 sec). In the final phase, the speaker was hidden by the black screen and only the listener was visible. The position of the speaker and the listener in the movies was counterbalanced between participants. A total of 8 movies were constructed by exchanging the positions of the speaker and the listener and by changing the combinations of speaker and the listener and speaker and the linguistic register. In addition to the movies for the test trials, we created movies for the pretest trials. These comprised the final phase of the test trial movies, that is, the cuts of the listeners only.

#### Procedure

The toddlers sat on their mothers’ laps or alone on a chair at a 70 cm distance from a 32-inch display (SONY KDL-32HVX). Stimulus presentation was controlled by PowerPoint (Microsoft); each stimulus was 25 cm × 34.5 cm in size on the display.

There were 2 pretest trials and 4 test trials. After the participant looked away from the screen for more than 2 sec, the next trial started. In the pretest trials, the toddlers were shown the infant or the adult listener in the final phase of the test trials to ensure visual preference toward each listener. By conducting the pretest trials, we intended to exclude the possibility that any difference in looking time in the test trials would be derived from a default preference for one listener. In the test trials, toddlers were shown 2 movies in each of the two register conditions: ADS condition: formal ADS to the infant/ formal ADS to the adult; IDS condition: IDS to the infant/ IDS to the adult). The speaker of each register was fixed, and the presentation order was managed so that the same register was not presented twice in a row. The experiments were recorded, and toddlers’ looking times were calculated offline by a first coder. In order to confirm inter-rater reliability, 25% of all responses were re-coded offline and under silent conditions by a second coder who was naïve to the hypothesis. The Pearson-product moment correlations of the first coder’s coding ranged from .99 to 1.00, with a mean of .99. Statistical analyses were conducted using R (version 3.3.3).

### Results

First, we analyzed the looking times of the pretest trials. The toddlers’ looking time to the infants was 10.63 sec (*SD* 5.60) and their looking time to the adult was 10.94 sec (*SD* 6.88). A paired *t*-test found no significant difference in looking times between the infant listener and the adult listener, *t* (15) = .22, *p* = .827, *d* = .06. This indicated that the toddlers did not differ in their default looking times to the adult or infant listener.

**Fig 2:**
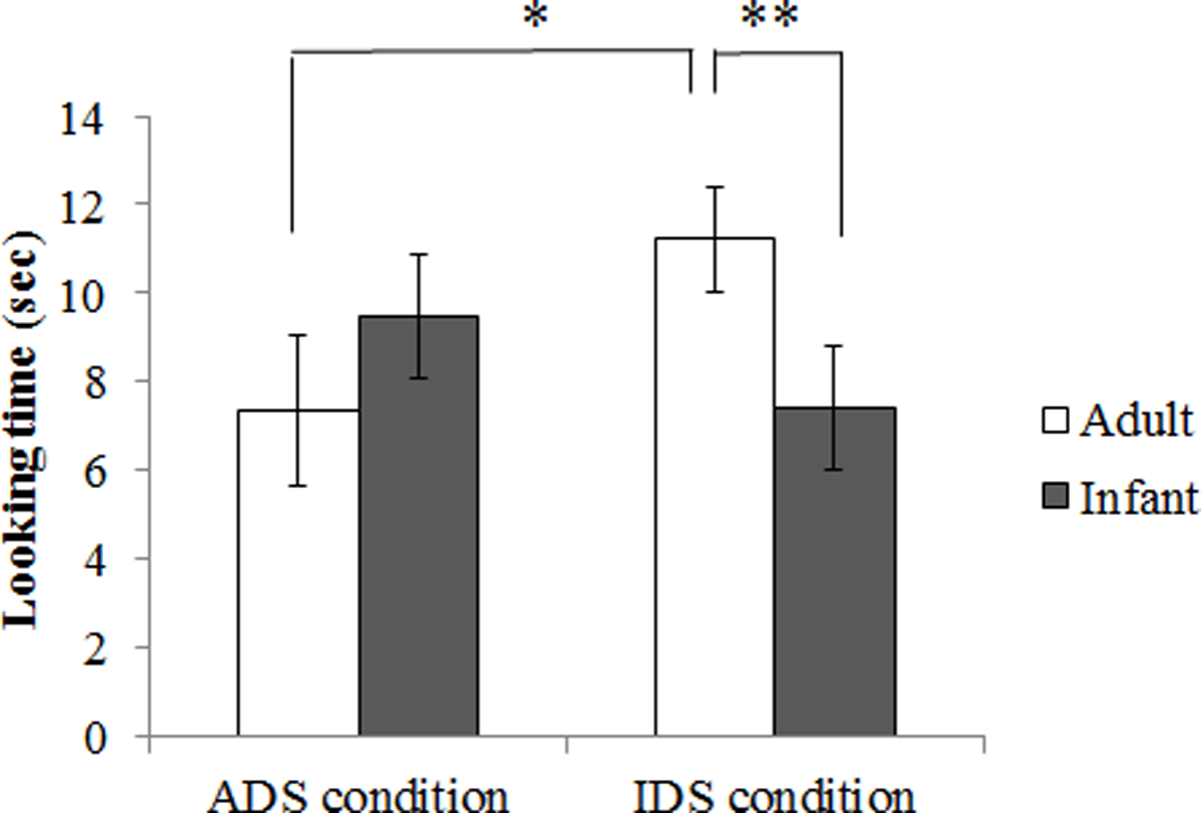
Mean looking times of ADS, and IDS condition in Experiment 1. Error bars indicate standard errors. ** *p* < .01.

In the main analysis, a 2 (condition: ADS, IDS) × 2 (listener: infant, adult) ANOVA revealed a significant interaction between condition and listener, *F*(1, 15) = 27.46, *p* = .0001, *η*_*p*_^*2*^ = .65, but no significant main effects of condition, *F*(1, 15) = .46, *p* = .510, *η*_*p*_^*2*^ = .03, or listener, *F*(1, 15) = .62, *p* = .443, *η*_*p*_^*2*^ = .04. An analysis of simple main effects in the IDS condition revealed that toddlers looked significantly longer at the adult than at the infant (Shaffer, *p* = .008) while there were no significant differences in the ADS condition (*p* = .102). Furthermore, toddlers looked significantly longer at the movie in which the adult was talked to in IDS versus ADS (*p* = .021) while there were no significant differences when the infant was talked in ADS and IDS (*p* = 0.151) (Fig 2 and S1 Data).

### Discussion

The results of the test trials suggest that 20-month-olds were partially successful in understanding adult sociolinguistic rules. The 20-month-olds looked longer at the listeners when the speakers spoke in IDS to adults compared to when they spoke in IDS to infants. However, 20-month-olds’ looking time did not differ when the speakers spoke ADS to infants and to adults. This suggests 20-month-olds thought that speaking IDS to adults was strange, whereas they did not think that it was strange to speak formal ADS to infants. These results cannot be explained by a simple visual preference for the listener because the results of the pretest trials revealed that the toddlers showed no preference between the infant and the adult. Therefore, knowledge about the relationship between a specific register and the listener is still immature although 20-month-olds have begun to understand this.

## Experiment 2

The results of Experiment 1 suggest that 20-month-olds have begun to grasp some register-listener relationships. In Experiment 2, we tested toddlers’ more abstract understanding of register. Specifically, we examined the factors that toddlers consider to be determinants of speech change. To examine whether toddlers under 2 years of age understand register change based on listeners, not on speakers, we used an infant-controlled habituation paradigm with a switch design [43–46]. This method makes it possible to isolate the factors that the toddlers associated with the register. After being habituated to two different movies in which an adult talks to an infant in IDS and an adult in formal ADS, the toddlers were given 4 test trials. The first was a baseline trial in which they saw the same movie as in the habituation phase. In the other test trials, either the register, the speaker, or the listener in the baseline movie switched to that of another of the habituation movies. From the standpoint of appropriate register use, the change of speaker followed the sociolinguistic rule, while the changes of register and listener did not. If toddlers associate registers with the listener, their looking time should be longer in the register-change and listener-change condition than in the baseline condition and should show no difference between the speaker-change and the baseline conditions. If toddlers associate registers with the speaker, their looking time should be longer in the register-change and speaker-change conditions than in the baseline condition and should show no differences between the listener-change and the baseline conditions. If they ignore the register and associate the listener with the speaker, their looking time should be longer in the listener-change and speaker-change conditions than in the baseline condition and should show no differences between the register-change and the baseline conditions. And if they associate the register, speaker, and listener, their looking times should be longer in the register-, listener-, and speaker-change conditions than in the baseline condition.

### Methods

#### Participants

Experiment 2 was conducted at Kyoto University. Another 16 20-month-olds (M = 1;7:29; range = 1;7:23–1;8:16) were recruited in the same manner as in Experiment 1. Twelve other toddlers participated in the study but were excluded from analysis because of fussiness and inattention (3), spontaneous recovery (6), and technical error (3).

#### Stimuli

One movie was prepared for pre- and post-tests. In the movie, one Japanese-speaking man faced the camera and waved his hand, then spoke to the toddler. For the main test, we presented the 8 movies, which were the same as the first phase of Experiment 1 except for three points. First, the listener was not hidden by a black screen and was visible to the participants. Second, the colors of clothes changed to grayscale because a preliminary experiment showed that vivid colors were too attractive for toddlers and weakened their attention to the utterances. Third, the lines that the speakers uttered were shortened (7 sec; IDS: “Hajimemashite, Konnichiwa. Onamae oshietekurerukana?” [Hi, nice to meet you. What’s your name?]; formal ADS: “Ohatsu ni ome ni kakarimasu. Onamae o oshiete itadakemasudesyouka?” [It’s a pleasure to meet you. Could you give me your name, please?]). Acoustic features of the utterances are as below: mean pitch was higher for IDS (M = 342.71 Hz) than for formal ADS (M = 313.38 Hz), pitch range was larger for IDS (M = 338.01 Hz) than for ADS (M = 257.74 Hz), and mean speech rate per mora was longer for IDS (M = 0.19 sec) than for formal ADS (M = 0.13 sec). The position of the speaker and the listener were counterbalanced between participants. All movies were 7.0 sec in duration.

#### Procedure

The toddlers sat on their mothers’ laps or alone on the chair 70 cm away from the 32-inch monitor (SONY KDL-32HVX). We used an infant-controlled habituation paradigm with a switch design [46]. The presentation of the movies (each 24 cm × 32 cm on the display) was controlled by the Habit X program [47]. The use of this software made it possible for a first coder to calculate toddlers’ looking times online and to automatically initiate test trials based on the predetermined criterion of their looking times during habituation.

**Fig 3:**
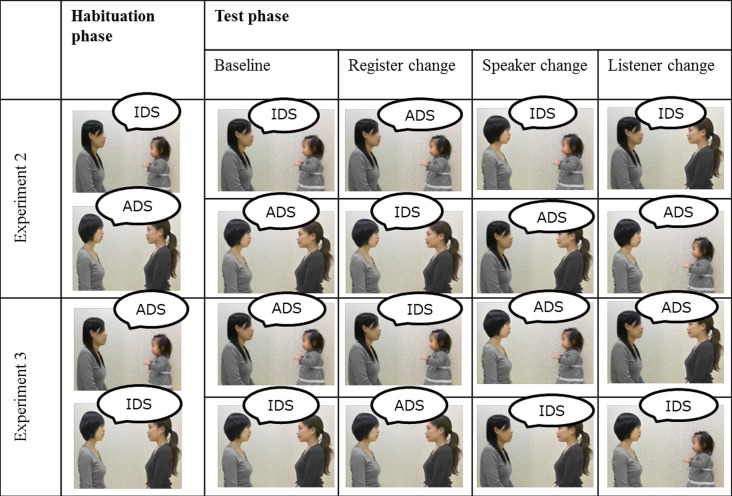
Examples of stimulus presentation in Experiment 2 and 3. During the habituation phase, the toddlers were shown two movies in semi-random order, with the same movie not occurring on more than 3 consecutive trials. After their looking time reached the habituation criterion, the test phase began, in which the toddlers were shown 4 movies. The 2 presentation patterns in the test phase differed depending on the baseline condition.

The presentation sequence is illustrated in Fig 3. In the habituation phase, toddlers were shown two different movies in which an adult talked to an infant in IDS and an adult in formal ADS. The combination of the register, listener, and the speaker was fixed during habituation, and the presentation order of the two movies was counterbalanced between participants. When total looking times on the most recent 3 consecutive trials fell below 50% of the total looking times on the first 3 trials or the number of trials reached 20, the test trials began. Toddlers were given 4 test trials. The movie of the baseline trial was identical to one of the habituation phases. On the other trials, the register, speaker, or listener paired with the baseline trial was switched. That is, in the register-change condition, while the combination of speaker and listener was the same as in the baseline condition, the register was switched from the baseline condition. Similarly, in the speaker-change condition, the combination of register and listener was the same as the baseline condition, but the speaker differed from the baseline condition. In the listener-change condition, the combination of register and speaker remained the same as the baseline condition, but the listener was switched. Thus, there were two presentation patterns based on the baseline trial. The order of the test trials was counterbalanced between participants, although the register change condition was fixed as the second trial. A trial continued until the toddlers look away from the display for more than 1 sec or the movie repeated 4 times (i.e., a maximum of 28 sec). Before the habituation phase and after the test phase, toddlers received pre-test and post-test trials to confirm whether or not the decrease of looking time during the test phase was due to fatigue. An attention-getter movie was also presented between trials.

#### Analysis

In a preliminary analysis, we made the following decisions about including or excluding data for the main analysis: (1) When the toddlers looked at the movie during the post-test for less than 25% of their looking time at the pre-test, they were regarded as being tired and excluded from analysis. (2) If the number of habituation trials reached 20 and the test trials began before the habituation criterion was reached, this was regarded as a failure to habituate, and the data were excluded from analysis. (3) If the toddlers’ average looking time on the most recent consecutive 2 trials and baseline test become over 50% of the total looking time on the first 3 trials, this was regarded as spontaneous recovery, and the data were excluded from analysis. The main analysis was conducted using looking time in the test phase. A second coder re-coded 25% of all responses, as described in Experiment 1. The Pearson-product moment correlations of the first coder’s coding ranged from .92 to 1.00, with a mean of .97.

### Results

The mean number of habituation trials was 12.19 (*SD* 4.81). To examine the effect of fatigue, we compared looking times at pre-test and post-test and looking times on the last habituation trial and post-test (Table 1). A paired t-test showed no difference between pre- and post-test, *t* (15) = 1.35, *p* = .20, *d* = .35, but looking time on the last habituation trial was significantly shorter than that at post-test, *t* (15) = -14.95, *p* < .001, *d* = 3.86. Thus, the decrease in looking time was not due to fatigue but to habituation.

**Table 1:** Mean (SD) looking times at pre- and post-test and on last habituation trial in Experiments 2 and 3.

	Pre-test	Post-test	Last habituation trial
**Experiment 2**	26.47 (4.01)	25.32 (4.91)	5.66 (2.27)
**Experiment 3**	27.1 (2.40)	24.59 (6.84)	4.56 (2.93)

**Fig 4:**
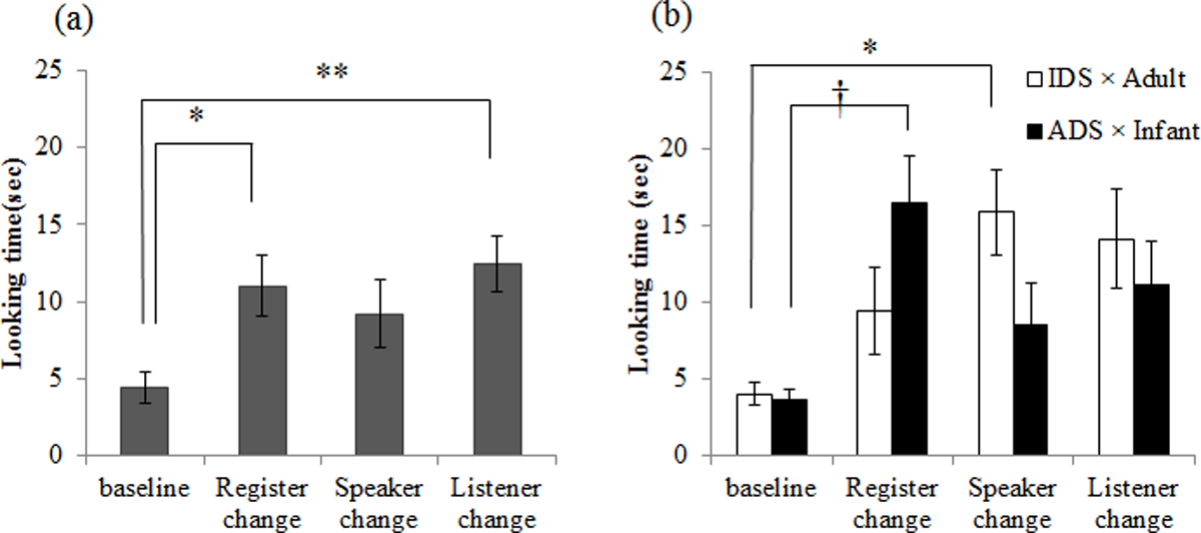
Mean looking times to baseline, register-, speaker-, and listener-change conditions in Experiment 2 (a) and 3 (b). Error bars indicate standard errors. * *p* < .05, ** *p* < .01.

A 2 (baseline condition: IDS to the infant, ADS to the adult) × 2 (order: speaker change first, listener change first) × 4 (test trials: baseline, register change, speaker change, listener change) ANOVA yielded a significant main effect of test trials, *F*(3, 36) = 4.86, *p* = .006, *η*_*p*_^*2*^ = .29. There were no other main effects and interactions. Multiple comparisons of test trials showed that the toddlers looked significantly longer at the register-change and listener-change conditions than at the baseline condition (Shaffer, register-change: *p* = .032; listener-change: *p* = .016), while there was no significant difference between the baseline and the speaker-change conditions (*p* = .259) (Fig 4A and S1 Data).

### Discussion

The results of Experiment 2 showed that 20-month-old toddlers dishabituated in the register- and listener-change conditions but not in the speaker-change condition, suggesting that they associated register with the listener, but not with the speaker. These findings appear to indicate that 20-month-olds understand that changes in register between IDS and formal ADS depend on listeners.

However, there are other possible interpretations of these results. One possibility is that the difference in looking times was derived from IDS preference, because infants generally prefer IDS to ADS [48,49]. In order to exclude this possibility of an effect of IDS preference, we focused on toddlers’ looking time to IDS and ADS. We selected the toddlers’ looking times to speaker- and listener-change conditions to conduct a 2 (baseline condition: IDS to the infant, ADS to the adult) × 2 (order: speaker change first, listener change first) × 2 (register: ADS, IDS) ANOVA. We found no main effects or interactions, suggesting that the results did not reflect an IDS preference in the toddlers.

A comparison of the results of Experiments 1 and 2 suggests that there is a contradiction. That is, although the toddlers in Experiment 2 succeeded in demonstrating an appropriate understanding of the register-listener relationship, the toddlers in Experiment 1 failed to do this. This contradiction may be explained by the difference in the duration of stimuli presentations before the test trials. The toddlers in Experiment 2 were presented with the habituation stimuli repeatedly, while the toddlers in Experiment 1 were shown the test stimuli without habituation. Thus, it may have been easier for the toddlers in Experiment 2 than for those in Experiment 1 to figure out the rule of the register that they had to use. On the other hand, the toddlers in Experiment 1 failed to use the rule because their understanding of individual rules of the register, especially the association between formal ADS and adult listeners, might not have been robustly developed. However, this gives another possible interpretation the results. That is, the toddlers may not have responded correctly to the switched movies based on an acquired understanding of a register, but rather, they may have learned the abstract rule (i.e., register change based on the listeners) or a specific rule during the habituation phase (e.g., the girl should be spoken to in IDS). We conducted Experiment 3 to exclude this possibility.

## Experiment 3

To exclude the possibility that the toddlers learned the abstract rule during the habituation phase, toddlers in Experiment 3 were presented with movies in which the combinations of registers and listeners were switched. Four test movies were presented similar to Experiment 2, after the toddlers were sufficiently habituated. If toddlers had learned the register-listener rule during the habituation phase, it was expected that their looking patterns would be identical to those in Experiment 2. However, if they had learned certain other rules, their looking times were expected to be longer for these other patterns compared to the result of Experiment 2. If they had learned associations among the register, speaker, and listener, their looking times were expected to be longer in the register-, listener-, and speaker-change conditions than in the baseline condition. If they had learned associations between the listener and the speaker, their looking times were expected to be longer in the listener-change and speaker-change conditions than in the baseline condition, and they were expected to show no differences between the register-change and baseline conditions. Moreover, if toddlers’ looking time were longer in the register-change and speaker-change conditions than in the baseline condition, and they also display no differences between the listener-change and baseline conditions, this would suggest two learning possibilities: toddlers learned the associations between the register and the speaker; or because they heard another version of ADS and IDS in the register-change and speaker-change conditions, they only learned acoustic stimuli that they did not hear during the habituation phase. Thus, if the looking time for one of the register- and speaker-change conditions were longer than the baseline condition, it would indicate that toddlers only learned ADS or IDS presented during the habituation phase. However, if only the looking time for the listener-change condition were longer than the baseline condition, this might reflect toddlers’ preference for the listener, but not indicate learning because there was no element of learning. Moreover, if their looking time showed no significant differences between the baseline and other test conditions, this would suggest that they did not learn any rule in the habituation phase.

### Methods

#### Participants

Experiment 3 was conducted in NTT Communication Science Labs. Sixteen 20-month-olds (M = 1;7:24; range = 1;7:0–1;8:24) who were recruited by NTT Communication Science Labs participated. Six additional toddlers participated but were excluded from analysis because of fussiness and inattention (4), spontaneous recovery (1), and failure to habituate (1).

#### Stimuli

Stimulus movies were the same as those used in Experiment 2.

#### Procedure

The procedures were the same as those in Experiment 2 except that the stimuli were presented on a 19-inch monitor (Mitsubishi RDT19IS) and an inconsistent combination (i.e., talking to the infant in formal ADS and talking to the adult in IDS) was used during the habituation phase (Fig 3). A second coder re-coded 25% of all responses, as described in the previous experiments. The Pearson-product moment correlations of the online coding ranged from .94 to 1.00, with a mean of .98.

### Results

The mean number of habituation trials was 8.31 (*SD* 3.18). As in Experiment 2, we examined the effect of fatigue by comparing looking times of pre- and post-test and looking times on the last habituation trial and post-test. Paired t-tests again showed no difference between pre- and post-test, *t* (15) = 1.85, *p* = .08, *d* = 0.48, and significantly shorter looking times on the last habituation trial than on the post-test, *t* (15) = -10.49, *p* < .001, *d* = 2.71, indicating that the decrease in looking time was due to habituation rather than fatigue.

A 2 (baseline condition: IDS to the adult, ADS to the infant) × 2 (order: speaker change first, listener change first) × 4 (test trials: baseline, register change, speaker change, listener change) ANOVA yielded a significant main effect of test trials, *F*(3, 36) = 6.68, *p* = .001, *η*_*p*_^*2*^ = .36 and a significant interaction between baseline condition and test trials, *F*(3, 36) = 3.20, *p* = .035, *η*_*p*_^*2*^ = 0.21. An examination of simple main effects of baseline condition on test trials revealed that baseline condition affected the looking times in test trials (IDS to the adult: *F* (3, 18) = 4.72, *p* = .013, *η*_*p*_^*2*^ = 0.44; ADS to the infant: *F* (3, 18) = 5.18, *p* = .009, *η*_*p*_^*2*^ = 0.46). Multiple comparisons revealed that the toddlers who were shown the stimuli in which the adult spoke IDS to the adult at baseline condition looked significantly longer at the speaker-change condition than at the baseline (*p* = 0.027), while the toddlers who were shown the stimuli in which the adult spoke ADS to the infant at baseline condition looked marginally longer at the register-change condition than at the baseline condition (*p* = .053) (Fig 4B and S1 Data).

### Discussion

The looking pattern in Experiment 3 differed from that in Experiment 2, suggesting that the participants failed to learn the rule of register and listener during the habituation phase. There was a significant interaction between the baseline condition and the test trials in Experiment 3, suggesting that the toddlers appear to have learned something during the habituation phase because they dishabituated in particular test trials. The toddlers that saw the speaker using IDS to the adult in the baseline condition dishabituated in the speaker-change condition; whereas the toddlers that saw the speaker using ADS to the infant in the baseline condition dishabituated in the register-change condition. Therefore, the toddlers in the two conditions seem to have learned different things, but what they learned was similar in that they dishabituated when presented with another version of IDS that they did not hear during the habituation phase. Thus, we inferred that they learned only IDS presented during the habituation phase. Taken together, the results of Experiment 3 suggest that although toddlers could learn something from the habituation phase, they could not learn the association between the register and the listener in the short time. Given that toddlers did not learn the rule regarding the register and the listener in Experiment 3, it is reasonable to assume that their looking pattern in Experiment 2 was caused by pre-acquired knowledge about registers. The results of Experiments 2 and 3 thus suggest that 20-month-old toddlers may already possess an abstract understanding of register; specifically, that a change in register depends on the listener, not on the speaker.

## General discussion

To elucidate the developmental process of register acquisition, one of the important topics in child development, we focused on IDS and formal ADS to examine whether toddlers under 2 years of age understand the relationship between the register and the listener. In Experiment 1, we found that 20-month-olds considered it irregular for people to speak IDS to adults, but they did not find it irregular for people to speak to infants in formal ADS. That is, although they expected that IDS would be spoken to infants, they thought that infants are not always spoken to in IDS. In Experiments 2, we demonstrated that 20-month-old toddlers associated a register with the listener and not with the speaker, suggesting that they understood that the register changes depend on the listeners, and not on the speakers. Experiment 3 supported the conclusion that the results of Experiment 2 reflected the toddlers’ already-acquired knowledge about the register, and demonstrated that it is difficult for toddlers to learn the register rules in the short time such as during the habituation phase. Additionally, our results indirectly indicate the possibility that 20-month-olds distinguish register from dialect because they do not associate register with the speaker. The present study thus provides the first evidence that 20-month-olds learn communication rules such as register, although their understanding is immature in the sense that they have not completely learned appropriate register-listener associations. Previous studies examined children over 3 years of age using more demanding tasks that required a certain level of language [14,16]. Thus, the present study extends these findings to show that the understanding of register begins at a younger age.

This study also revealed an asymmetry in understanding the register of IDS and formal ADS in toddlers. That is, for toddlers, talking to adults in IDS is strange, but talking to infants in formal ADS is not strange. Why is there such a difference? One possibility is that asymmetry might have occurred because of differences in the frequency of encountering dialogue situations, especially because it is not very unusual to speak to infants in ADS. Not all adults are accustomed to speaking IDS, and some caregivers avoid speaking IDS to their children. In other words, although it is rare to encounter a situation in which adults are spoken to in IDS, it may be less rare to encounter a situation in which toddlers are spoken to in ADS. Considering that eavesdropping on other people’s conversations may be the source of the learning of register [14], it is plausible that the difference in frequency of encountering dialogue situations affects children’s understanding of register. In addition, while most toddlers regarded talking to toddlers in formal ADS as not strange, there were others who did regard talking to toddlers in formal ADS as strange. Identifying differences in the language environments of individual toddlers may be helpful in clarifying the learning source of register and the developmental trajectory of register acquisition.

Another possibility is that toddlers attend more to IDS and attend less to ADS. Many studies have demonstrated that infants prefer to listen to IDS than ADS [6]. Moreover, the result of Experiment 3 showed that toddlers learn IDS, but not ADS. Therefore, it is possible that attention allocation of infants’ is asymmetrical. As a result, there may be a time lag in learning the rules of each register.

The toddlers in Experiment 1 failed to use the rule of association between formal ADS and an adult listener. However, the toddlers in Experiment 2 and 3 showed that they already have more abstract knowledge. That is, the toddlers understood the rule that register changes depend on listeners and not on speakers. The result of Experiment 3 demonstrated a certain learning effect. Therefore, it is possible to conclude that the association between formal ADS and adult listeners became clearer and robust by repeated stimuli presentation in Experiment 2. There are more specific rules than abstract rules, and therefore, it is plausible that there is a time lag in understanding. Moreover, it is not strange that understanding abstract rules is slower than understanding specific rules.

One limitation of this study is that we could not identify the elements of IDS or formal ADS that toddlers associated with a register because we used stimuli having acoustic, lexical and statistic characteristics. In the development of understanding register, we should remember that children might learn acoustic modification first, and lexical and syntactic modifications next, as proposed by Anderson (1999) [50]. Given previous evidence that IDS preference in 4-month-olds is determined by pitch range [51], it is possible that 20-month-olds associate a certain acoustic characteristic with IDS. However, it is also possible that younger children notice lexical and statistical characteristics of the register. Previous studies reported that 2- to 3-year-olds use more diminutives (e.g., *Kay-Kay* for *Kate*), which is one type of words used in IDS [9] and 3- to 5-year-olds use have a shorter mean length of utterance (MLU) when they talk to infants [10]. Considering that understanding of register precedes its production, the association of lexical and statistical modification with register may begin a bit earlier than 2 to 3 years. Uncovering this issue will help us to understand the acquisition of register.

As mentioned in the introduction, around 2 years of age is the period when toddlers’ language skills develop dramatically. Toddlers in this period become able to speculate referents of unknown words accurately using function words [52] and trivial linguistic cues such as fluency [53]. Moreover, they become able to learn words in complex situations such as adults’ conversations [54]. With these cognitive developments and this expansion of learning opportunities, toddlers experience a vocabulary spurt, and their syntactic skill also develops [32,33]. Evidence from this and previous studies suggests that toddlers around 2 years old seem to be learning not only lexicon and syntax but also communication rules such as register in order to communicate with other people. It means that they also realize that a speech act involves more than a simple exchange of information. Incomprehension of lexicon and syntax can cause a failure of communication, whereas incomprehension of register does not. Therefore, it is surprising that toddlers pay attention to such rules during the early stages of acquiring a lexicon and syntax.

The present results suggest a number of possibilities for future studies. First, it is necessary to manipulate the speaker and change the relationship between the speaker and the listener. Although our study manipulated only the listener, register use changes based on the relationship between the speaker and the listener. That is, a speaker speaks in various ways, but a listener is also spoken to in various ways, depending on his or her relationship with the speaker. Thus, it is necessary to manipulate the relationship between the listener and speakers of different status. Second, it would be valuable to examine children’s understanding of other registers, such as casual ADS, foreigner talk that is used to a beginner of the language, and the manner of speaking used by a person in authority to subordinates. Third, as they grow, children develop not only their language skills but also other aspects of cognitive ability, such as context sensitivity and cognitive flexibility. Thus, examining the interaction between the understanding of register and other abilities may reveal other aspects of the process of learning register. Additionally, it is also interesting to clear differences and similarities between register change in one language and code-switching, which is a shift among several languages　 based on listeners and situations.

In summary, we found that even toddlers under 20 months of age understand that people change their way of speaking depending on the listener. However, their understanding of the relationship between register and listener is immature, and more time is needed for them to fully understand the appropriate use of a register. That is, 20-month-old toddlers understand that there are sociolinguistic rules of dialogue before they learn linguistic markers of registers and appropriate combinations of register and listener.

## Supporting information

S1 Data Individual data of looking times during test phase, pre- and post-test, and last habituation trial, and number of habituation trials in each experiment. (XLSX)

S1 Supporting information (DOCX)
