# Supplementary material for: Sensitivity to linguistic register in 20-month-olds: Understanding the register-listener relationship and its abstract rules
Source: PLoS One. 2018 Apr 9;13(4):e0195214. doi: 10.1371/journal.pone.0195214 (PMC5891006; doi:10.1371/journal.pone.0195214)
Supplement: S1 Supporting information — (DOCX) [file pone.0195214.s002.docx]

**Supporting Information: Experiment 4**

Some readers may assume that habituation in Experiments 2 and 3 was not required and the test phase alone was sufficient. To dismiss this idea and confirm that the paradigm used in Experiments 2 and 3 was appropriate, we conducted Experiment 4. In this study, after habituation was shaped to keep toddlers’ conditions the same as in Experiments 2 and 3, the toddlers were presented with a test phase that used new movies, that is, habituation was violated. If they fail to show the same looking pattern as Experiment 2 when habituation is violated, this is evidence that the habituation-switch method is an appropriate method for examining the toddlers’ understanding of register.

**Methods**

**Participants**

The experiment was conducted in NTT Communication Science Labs from August 10, 2015 to March 15, 2016 as in Experiment 3. Sixteen 20-month-olds (M=1;8:11; range=1;8:2-1;8:25) were recruited in the same way as in Experiment 3. Nine other toddlers participated in the study but were excluded from analysis because of fussiness and inattention (7) and technical error (2).

**Stimuli**

Eight new movies were prepared, in addition to those used in Experiment 2. The format of the 8 new movies was same as the original movies, but the listeners were a new 15-month-old girl and a new woman.

**Procedure**

Procedures were same as in Experiment 2 (S1 Fig), except that the stimuli were presented on a 19-inch monitor (Mitsubishi RDT19IS), the movies used in the test phase were new movies, and presentation times were extended to a maximum of 112 sec. As in the other experiments, 25% of responses were coded by a second coder. The Pearson-product moment correlations of the on-line coding ranged from 0.98 to 1.00, with a mean of .99.


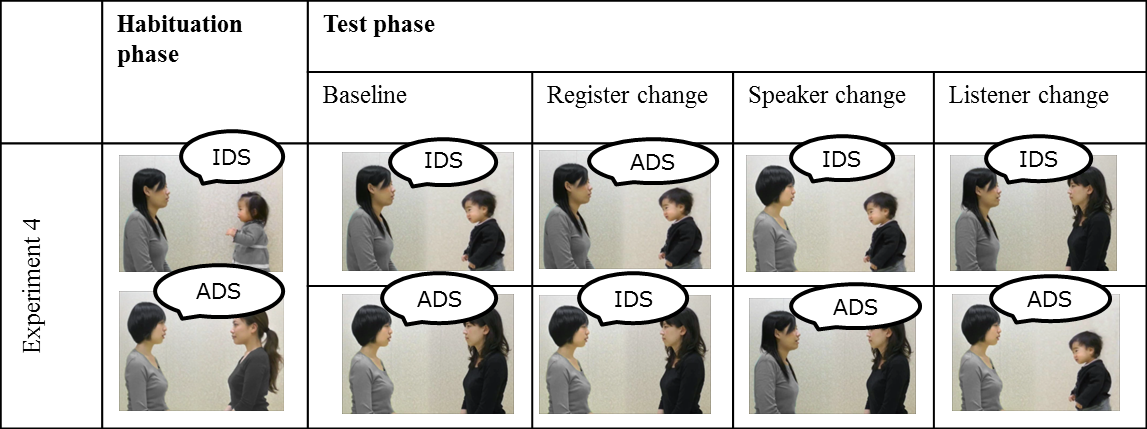


**S1 Fig. The example of stimuli presentation in Experiment 4.** During the habituation phase, the toddlers were shown two movies in semi-random order, with the same movie not occurring on more than 3 consecutive trials. After looking time reached the habituation criterion, the test phase began, in which toddlers were shown 4 movies. The 2 presentation patterns in the test phase differed depending on baseline condition.

**Results**

The mean number of habituation trials was 9.25 (SD 4.12). A paired *t*-test revealed that looking time was significantly shorter at post-test than at pre-test, *t*(15) = 5.28, *p* = .038, *d* = 0.61, and looking time was significantly shorter on the last habituation trial than at post-test, *t*(15) = -13.73, *p* < .001,*d* = -3.54 (S1 Table). Thus, although we cannot deny that the toddlers felt fatigue, the decrease in looking time was not due to fatigue but to habituation. A 2 (baseline condition: IDS to the infant, ADS to the adult)×2 (order: speaker change first, listener change first)×4 (looking time on test trials: baseline, register change, speaker change, listener change) ANOVA yielded a significant interaction between baseline condition and looking time on test trials, *F*(3, 36) = 9.45, *p* = .00001, *η_p_^2^* = .44. The examination of simple main effects of baseline condition on looking time on test trials revealed that the baseline condition affected looking times on test trials (ADS to the adult: *F*(3, 18) = 4.45, *p* = .017, *η_p_^2^* = .43; IDS to the infant: *F*(3, 18) = 6.02, *p* = .005, *η_p_^2^* = .50). Multiple comparisons revealed that the toddlers who were shown the stimuli in which the adult spoke ADS to the adult at baseline looked significantly longer at the speaker-change condition than at the listener change condition (*p* = .004) while the toddlers who were shown the stimuli in which the adult spoke IDS to the infant at baseline looked longer at the baseline than at the register-change and listener-change condition (*p* = .005) (S2 Fig).

**S1 Table. Mean (SD) looking times (sec) at pre- and post-test and on last habituation trial in Experiment 4.**

|  | **Pre-test** | **Post-test** | **Last habituation trial** |
| --- | --- | --- | --- |
| **Experiment4** | 27.69 (0.12) | 24.34 (5.45) | 5.36 (4.36) |


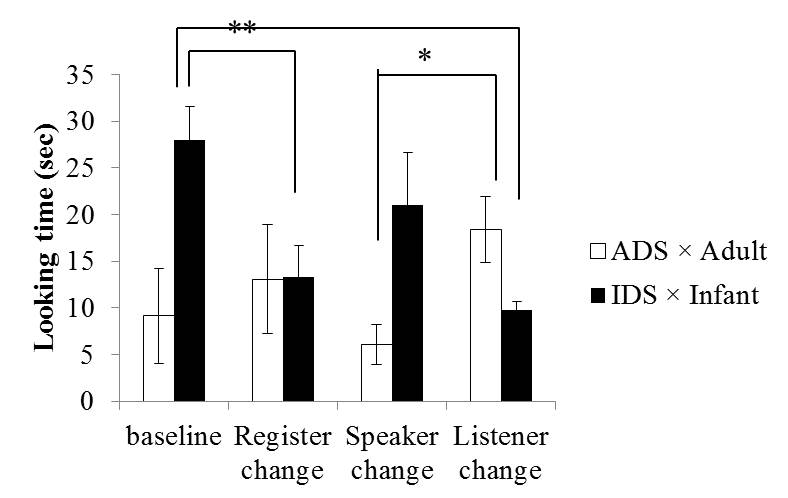


**S2 Fig. Mean looking times to baseline, register-, speaker-, and listener-change condition in Experiment4.** Error bars indicate standard error.

**Discussion**

The results of Experiment 4 showed a pattern different from that of Experiments 2 and 3. The result for toddlers who were shown stimuli with the adult speaking IDS to the infant at baseline suggests that the toddlers looked longer at the stimuli when the rule of register was maintained, while the result for toddlers who were shown stimuli with the adult speaking ADS to the adult at baseline indicates that the toddlers looked longer at the stimuli when the rule of register was violated. These contradicting results may have occurred because shaped habituation was violated in the test phase. Thus, to test toddlers’ understanding of register, presenting only the test phase was insufficient, and habituation was needed.
